# Comparison of two needle versus one needle lesioning techniques for thoracic medial branch neurotomy

**DOI:** 10.1016/j.inpm.2022.100085

**Published:** 2022-03-25

**Authors:** Richard Derby, Yakov Vorobeychik, Byron J. Schneider, Zachary L. McCormick

**Affiliations:** a Spinal Diagnostics and Treatment Center, Daily City, Ca, USA; b Penn State Milton S. Hershey Medical Center, Penn State College of Medicine. Department of Anesthesiology & Perioperative Medicine, Hershey, PA, USA; c Physical Medicine and Rehabilitation, Vanderbilt University Medical Center, USA; d Department of Physical Medicine and Rehabilitation, University of Utah School of Medicine, Salt Lake City, UT, USA

## Abstract

**Background and objectives:**

No prior studies have investigated an assumed advantage of creating a radiofrequency strip lesion for posterior element spinal axial pain using a two-needle technique (TNT) compared to a one-needle technique (ONT) that creates a single ovoid lesion. We explore the relationship of TNT compared to ONT on the magnitude and duration of pain relief following thoracic medial branch neurotomy (TMBN).

**Methods:**

This study is a retrospective audit of consecutive patients treated with TMBN at a single site and interventionalist over ten years (2007–2017). All patients had undergone TMBN after failed conservative care and, with few exceptions, patient-reported ≥ 70% pain relief after thoracic medial branch block (TMBB). All patients had TMBN performed with a medial to lateral (MLA) radiofrequency cannula approach using either an ONT or TNT technique. We used parametric and nonparametric statistics and three levels of case analysis to assess for intergroup differences.

**Results:**

Thirty-five consecutive patients underwent their primary TMBN, and two underwent both on a subsequent repeat using the MLA approach, 19 using the ONT, 18 using the TNT. The TNT group had clinically and statistically greater pain relief magnitude and duration than the ONT subgroup. The difference resulted in non-overlapping 95% confidence intervals for both percent pain relief and duration of pain relief using three levels of case analysis.

**Conclusion:**

The comparison of TMBN techniques demonstrates a statistically significant separation of TNT to ONT sample mean values for magnitude and duration of pain relief when using TNT compared to ONT for TMBN using an MLA.

## 1 Introduction

Denervating a painful anatomical structure will eliminate or significantly reduce index pain, but only if this structure is a source of pain and only if the denervation is complete. [1] The literature favors superior outcomes in the lumbar and cervical regions when one performs medial branch neurotomy (MBN) according to anatomical landmarks and when creating several larger lesions to help incorporate the medial branch within the lesion radius [2]. However, the method of achieving accurate denervation is a contentious topic, with proponents of a minimally invasive technique typically favoring narrow gauge needles placed adjacent to the medial branch using sensory stimulation. The proponents of using larger gauge needles favor multiple adjacent lesions or newer radiofrequency MBN devices that create larger radius lesions [3,4].

Using two radio-frequency cannulas placed side by side utilizing either a bipolar technique or two simultaneously heated unipolar radiofrequency canulae is an intuitive method of efficiently creating a larger lesion as an alternative to repositioning a single radiofrequency cannula. Different practitioners have used and described their version of TNT that they conceived of without knowing that others used it, and probably many more have conceived of and used the technique as a matter of routine practice [[5], [6], [7]].

Simultaneously heating electrodes placed parallel to each other should create a strip lesion that exceeds in size a summation of two separate lesions created by a single electrode [8]. In 2006 and 2010, Lee and Derby studied and described the TN technique in ex-vivo porcine spinal tissue using two parallel 20-gauge radiofrequency cannulas. Dr. Lee recorded lesion characteristics at 1–9 ​mm from the active electrode tips, comparing unipolar lesions to simultaneous lesions created by two parallel spaced needles (TNT) or (SURF). ^6 8^ Using the largest then readily available 20 gauge radiofrequency canulae, the investigators achieved maximal effective lesions at heating temperatures greater than 80° C for 90 ​s. Simultaneously heated, two needles could be placed 6 ​mm apart and achieve 60° C at the midpoint, compared to 40° C when heated separately. However, in a repeat investigation in cadaveric interspinous ligaments using the same 20 gauge radiofrequency cannula, the investigators found the maximal effective distance was 4 ​mm for simultaneously heated needles compared to 2 ​mm in sequentially heated radiofrequency needles [9].

In 2014 Cosman et al. studied radio-frequency lesion characteristics during a range of electrode separation differences using different RF needle gauges, electrode active tip lengths, and heating parameters. The investigators sought to achieve a consistent, effective lesion width between electrodes instead of the two overlapping ovoid-shaped lesions made by separate electrodes [10]. In the case of 18-gauge unipolar electrodes, a separation less than 7–8 ​mm at a temperature-time of 85C for 90 ​s will consistently reproduce a lesion comparable to using the bipolar technique at approximately 10 ​mm of electrode separation.

In the present study, our primary goal was to expand technical details of the TNT used for thoracic medial branch neurotomy (TMBN [8,9,11], and compare TNT treatment outcomes to one-needle-technique (ONT). We seek to reject the null hypothesis that there is no statistical difference in the magnitude and duration of pain relief following TMBN performed with TNT compared to ONT at a significance of p<.05. We secondarily explore each technique's mean differences and confidence intervals to assess clinical significance. Last, we explore the effect of potential confounding factors that may bias outcome.

## 2 Methods

This study continues our recently published thoracic MBN outcome study [11], expanding the time frame from 2010 to 2016 to 2007–2017 to add six earlier cases when the first author began using the TNT and transitioned to a medial-lateral approach (MLA) for TMBN. We chose the latter cutoff date to limit cases performed in a single facility setting for consistency, adding one case. We specifically confined the cases to those in which MLA was used to eliminate one potentially confounding variable; doing so required excluding only one case. All TNT procedures were performed using simultaneously heated unipolar electrodes rather than a bipolar technique except for a few initial cases. As a comparison paper, we do not define success. We use the patient magnitude of reported pain relief at six months following their first TMBN as the response variable, percent pain relief, and how long that degree of pain relief lasted as the response variable for the duration of pain relief.

Based on a programmed search using regular expressions, we identified patients using the stored dictated reports rather than current procedural terminology codes, allowing for the separation into cervical, junctional cervical-thoracic, thoracic, junctional thoracic-lumbar, and lumbar MBN procedures. We included only thoracic procedures and not junctional ones, except for two patients with MBNs performed at different sessions at the cervical or lumbar regions.

A diverse group of physicians referred the patients either for consultation for interventional treatment or specifically for consideration for TMBN. The first author considered patients' candidates for thoracic MBN based on the history & physical, imaging studies, and responses to prior treatments. Progression to MBN typically required robust response to diagnostic MBB with a minimum NRS reduction of 70%, as this cutoff best correlated with positive outcomes in his lumbar RF patient population [12]. However, on three occasions, the first author did matriculate patients not meeting his MBB cutoff based on clinical judgment; the reasons to do so for specific patients were described in our prior study [11]. The first author with over 25 years of interventional spine experience performed all MBN procedures at a single ASC facility.

We obtained IRB approval # IRB00012773 (Eck Institutional Review Board) as a retrospective audit, recognizing that there is no immediate benefit for the selected patients; however, study findings may provide better evidence-based patient consent for future patients and providers. This research did not receive any specific grant from funding agencies in the public, commercial, or not-for-profit sectors. No authors had any conflict of interest, and we conducted the study according to the Declaration of Helsinki.

## 3 Technique

We briefly described the medial to lateral approach using the ONT and TNT in our recent TMBN paper accepted for publication to *Interventional Pain Medicine* [11]. Foremost, all painful segments were typically addressed in one session. However, the first author performed each side unilaterally in sequential sessions in a few patients with bilateral pain at multiple levels.

**Fig. 1:**
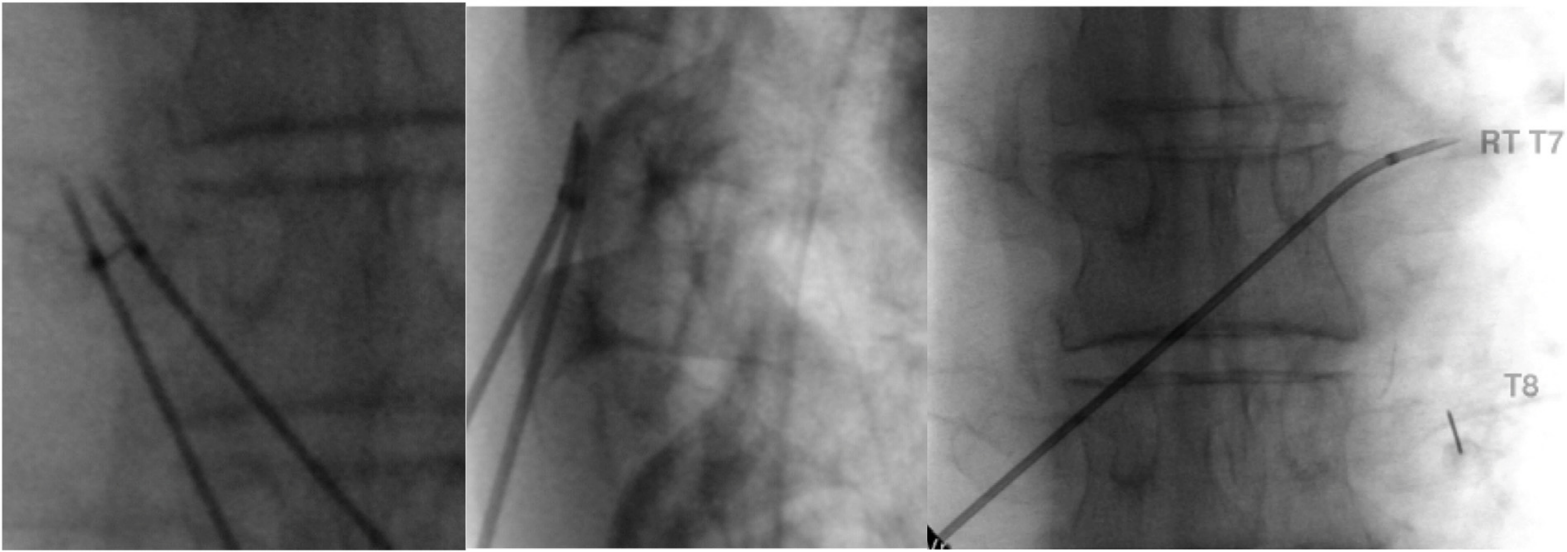
Less than ideal quality images, which is more the norm than the exception in the thoracic spine, the patient has had consistent 70–100% pain relief over the years following their first and repeat T7-T10 TMBN with complete return of the activities of daily living after each TMBN. The patient is one of the two cases having both ONT and TNT performed at sequential sessions. In addition to having accompanying lateral images, the AP images illustrate a 60-degree approach angle facilitating advancing the distal needle tips off the upper border of the transverse process staying within the confines of the lateral tip of the transverse process; the clinical purpose is to be closer to medial branches located between the transverse processes. The distance ∼5 ​mm between electrodes is easily within the distance to assure a uniform strip lesion between the electrodes with a 10 ​mm active tip; the electrode tips are safely medial to the lateral border of the transverse process. One can see a slight distal needle curve on the slightly rotated lateral image; the low angle of cannula insertion keeps the electrodes well away from the intervertebral foramen and relatively parallel to the intertransverse ligament. The right image is taken from the same patient's first TMBN several years earlier using an ONT which provided 70% pain relief for less than a year compared to 100% relief for more than a year using the TNT. Note on the far-right image the 1.5-inch 25-gauge needle and syringe, the tip on the T8 transverse process in position to inject 0.5 ​ml of 50% dextrose.

The ON and TN insertion techniques are the same (Fig. 1); however, using two needles allows placing the exposed electrodes side by side, heating simultaneously to create a strip lesion (Fig. 1, A3). In earlier cases, the first author inserted a single cannula medially, 0–∼30° parallel to the transverse process, one to two finger breaths contra-lateral to the target level (Fig A2-left). In later years the cannula was often inserted at or one to two interspaces below the target level, the entry points typically over or ipsilateral to the spinous process, the angle with the transverse process varying from approximately 40 to 60° (Fig. 1). The needles were typically advanced off the transverse process by several mm, but, at the time, not purposefully advanced further and always kept within the lateral boundary of the transverse process tip (Fig. 1).

For both the ONT and TNT, the first author performed a single lesion by heating the electrode(s) to 90° C for 90 ​s and supplemented all lesions with the addition of 0.5 ​ml 50% dextrose injected several mm above the mid to distal transverse process using one separate 25-gauge needle placed on the superior lateral aspect of the transverse process (Fig. 1 far-right). However, in some cases, the injection was done through one of the radiofrequency cannulas. Fifty percent dextrose was injected as a mild neurolytic to compensate for the probability that fibers of the medial branch may be located in the mid intertransverse space [13,14]. After the fact and for future consideration, we demonstrate advancing an RF cannula to the proximity of the midpoint of the intertransverse space in a cadaver. (Fig A4).

By comparing the distance between exposed canulae and the width of the transverse process on the A-P fluoroscopy images, we recorded and verified that separation distances were within 7 ​mm or less, allowing a continuous strip lesion for 18 gauge cannula during simultaneous heating [15]. (Fig A3).

## 4 Data and statistics

We programmatically and manually extracted the data directly from dictated reports and from a secured relational SQLite relational database containing parsed original physician reports and fluoroscopy images, using only not templated data. The tables contained ten key report header fields, including the type of service and procedure(s) performed during the encounter in addition to the unfiltered text version of dictated word document reports.

Confirmed by manual verification, several database queries identified all patients that had undergone a thoracic MBN within the study years. The query selected MBN(s) performed on thoracic medial branches, although a few records were junctional procedures that we manually excluded. The system facilitated our subsequent electronic and manual data gathering by quickly selecting and displaying all patient dictated reports and stored images.

We named the independent predictor variable (factor or group) “technique”. It has two potential predictor variables or subgroup levels, TNT and ONT. We selected index pain relief (PR) and pain duration (PD) as the dependent response variables, determining homogeneity (normal distribution) of dependent variables using a Leven Test (LT). In addition, we gathered potential confounding clinical and independent technical variables for preliminary assessment, realizing that eighteen factors with less than forty observations increase type 1 errors and may result in too few observations in some categorical subgroups to compare mean differences reliably.

We used a parametric one-way analysis of variance (OW-ANOVA) and a nonparametric Mann Whitney *U* Test (MWUT) to test the null hypothesis using PR and DR as dependent response variables (Table A1-4). Specifically seeking to show there is less than a 5% chance that the results of TNT and ONT come from the same population.

Statistical analysis and plotting were done using DataFrames.jl, HypothesisTests.jl, and StatsPlots.jl. The software are Julia packages designed for data manipulation, statistical analysis, and statistical plotting.

**Fig. 2:**
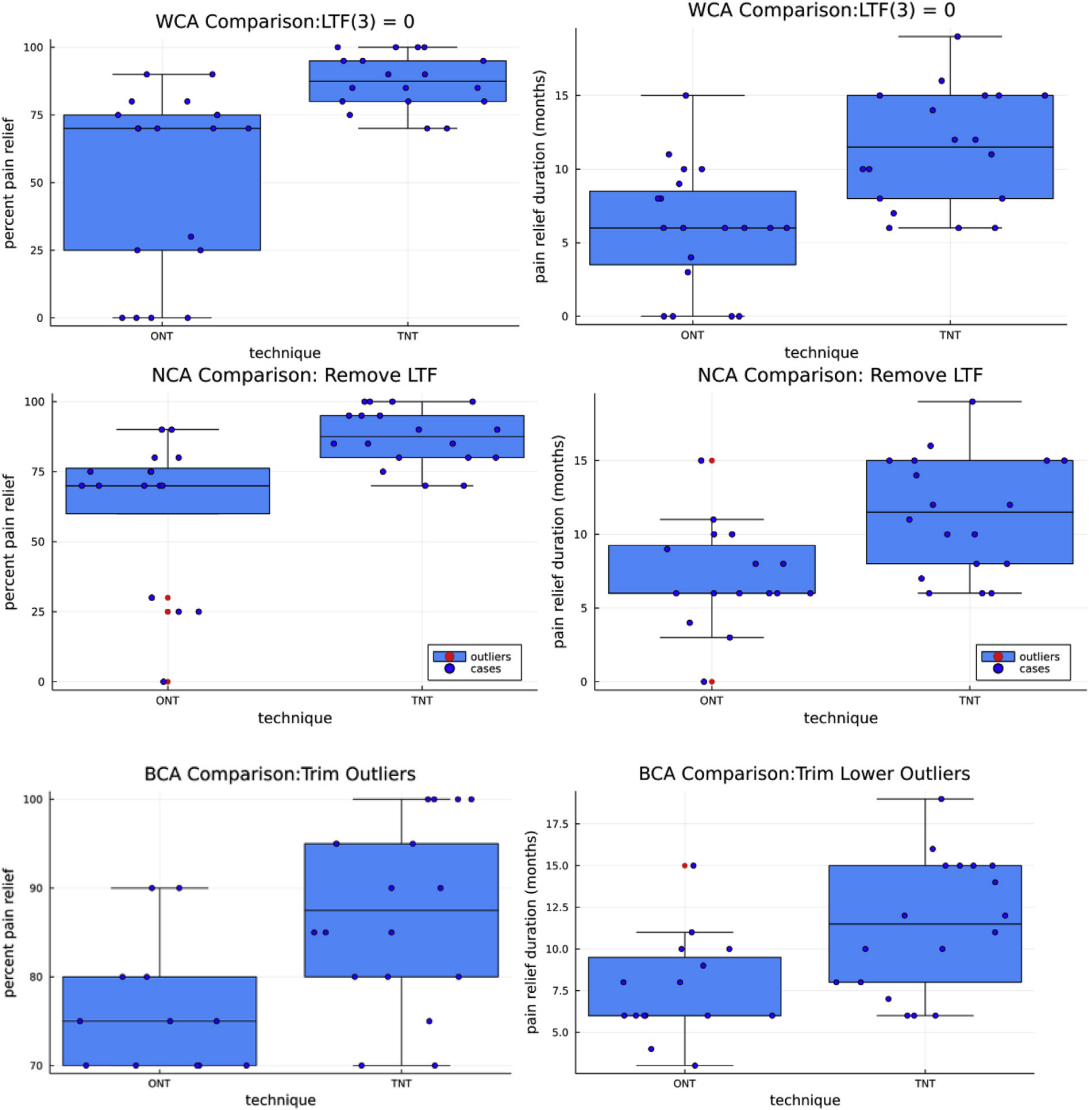
The blue boxes show the second and third quartiles; the black line within or on the border is the median value. The whiskers extending from the represent the variability of 1.5 quartile distance above the second and below the third quartiles. Outside the whisker, boundaries may be considered outliers. We use WCA, NCA, and BCA datasets from top to bottom to plot the degree of pain relief on the left and the duration of pain relief on the right. The NCA shows outliers on the same line as the outlier circles. Only the ONT has outliers, three below, one above, and no cases (black dots) variability below the third quartile; thus, there is no lower whisker, and the lower box black line is the median. In addition, we removed three LTF cases. Trimming the data of LTF and outliers only slightly changed the ONT results; the TNT having no outliers is unchanged. Please note the difference in pain relief and duration scales in the BCA. (For interpretation of the references to colour in this figure legend, the reader is referred to the Web version of this article.)

To limit potential bias when testing our TNT:ONT hypothesis, we used three trimming strategies named the following: worst-case analysis (WCA), that replaced lost to follow-up cases (LTF) with 0% relief of pain and 0-month duration of pain relief; neutral case analysis (NCA) that discarded the LTF cases; and best-case analysis (BCA) that trimmed the database outliers. A primary reason for multiple case analysis was the actual or chance occurrence that the ONT group contained all the LTF cases (3) and all the outliers, three lower and one upper (Fig. 2).

## 5 Results

**Fig. A1:**
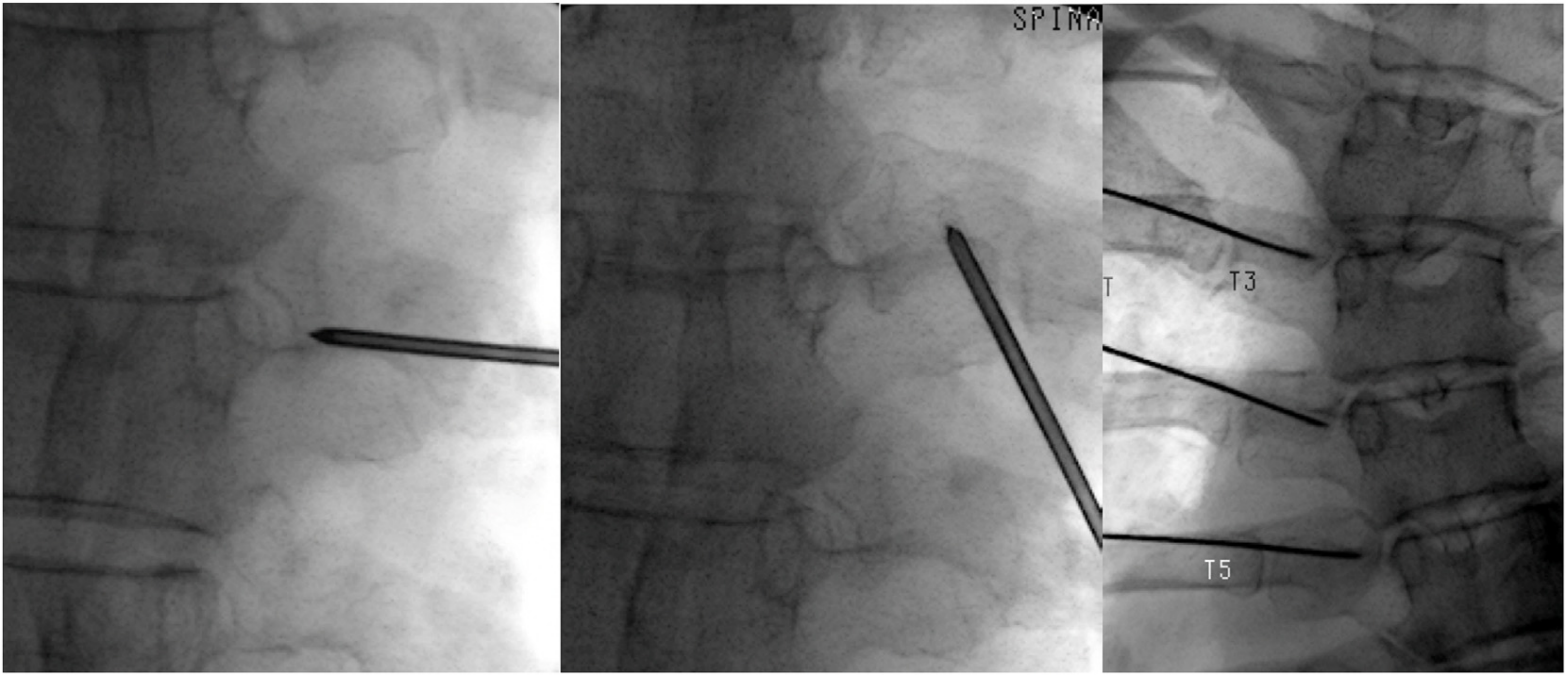
The center and left radiographs are images of the first author's first TMBN, approaching the target distal transverse process with LMA performed in 1999. At six weeks follow-up, the patient had relief of her index pain. We performed the procedure before the study starting date, and, therefore, we did not include the case in the study. The right image also shows a LMA with the then available 20-gauge RFC but within the starting study date. The patient reported greater than two years of significantly reduced pain before a repeat procedure became necessary. Performed within the 1/2007 starting study date and the only LMA after the date, we excluded the case because the approach was not an MLA.

**Fig. A2:**
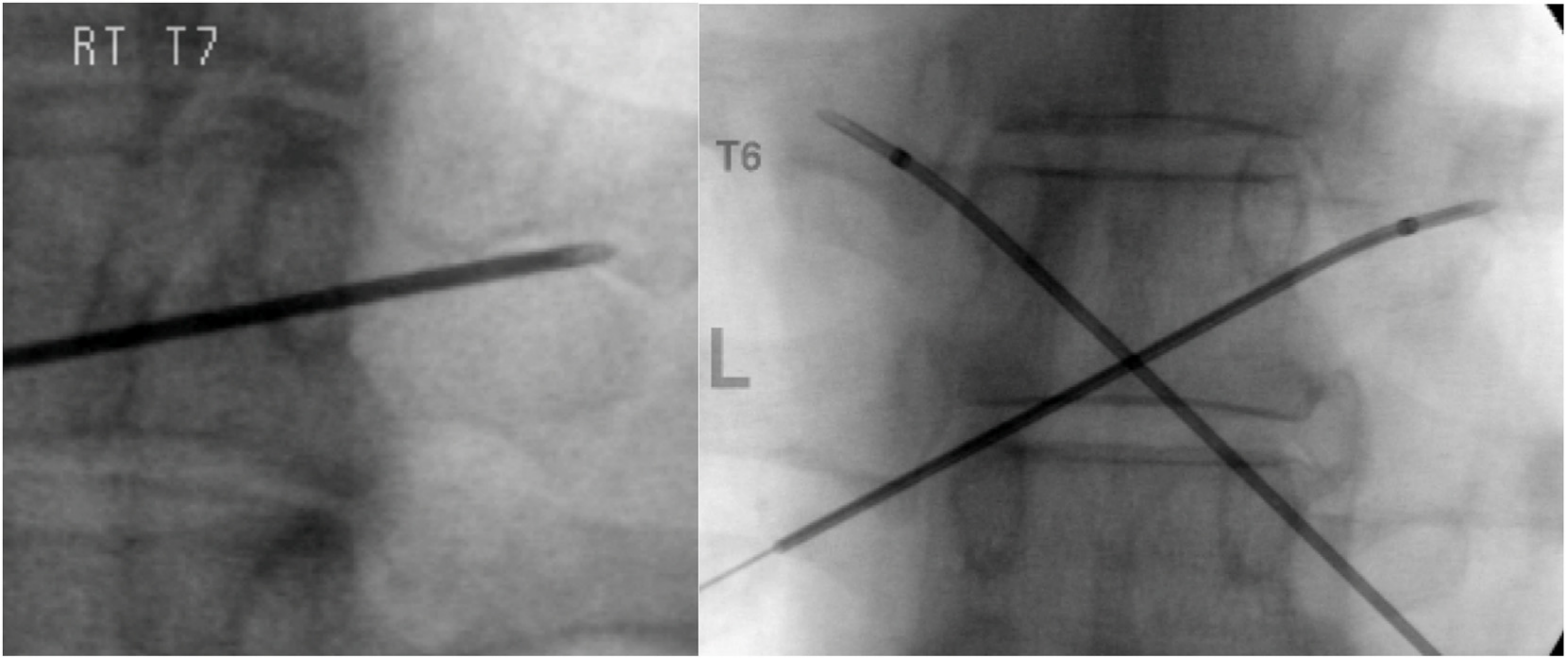
The left fluoroscopy image illustrates unilateral ONT performed using ∼20-degree canulae angle on a T7-T10 TMBN. The patient returned a year for asking for a repeat TMBN despite reporting only 30% but was satisfied and grateful for partial relief. We had matriculated the patient at their request, as their MBB relief was below our 70% cutoff; their results were consistent with the MBB results. The right image illustrates a bilateral ONT at ∼40° MLA insertion angle. The distal tip has a slight curve that facilitates directing the needle to contact the approximate base of the transverse process with the tip pointed downward. After contacting bone, one rotates the cannula 90° to achieve a more parallel course with the slightly upsloping process. The skin entry point is center to contralateral at lower needle angles and slightly ipsilateral for steeper angles and created when inserting the needle at one to two segments below. Our preliminary factor analysis suggests a mild association of better outcomes using steeper angles.

Our database search results resulted in a consecutive series of forty-two patients trimmed to thirty-five after eliminating six patients with junctional MBNs and the one with an LTM approach, resulting in a total of thirty-five patients (Fig A1 far right). We identified two patients that had undergone an ONT during their original TMBN but a TNT on a subsequent repeat (Fig. 1). Since a primary goal was to compare ONT to TNT, we included both as separate cases. Nineteen patients underwent TMBN using the ONT, eighteen using a TNT technique.

Both the magnitude and duration of pain relief were greater in the TNT compared to the ONT group using both WCA (p ​= ​.00015) and BCA (p ​= ​.00215) calculated using the parametric OW-ANOVA test (Table A1). The p-values were the same as the one-sample *t*-test, the square-root equivalent of the OW-ANOVA using two groups. Nonparametric hypothesis testing with the Mann Whitney *U* Test (MWUT) showed p-values < .0001 and .0047 for the magnitude of pain relief when using WCA and BCA, respectively. For the duration of pain relief using the OW-ANOVA test, we found p-values ​= ​.0008 and .0075 when using WCA and BCA, respectively (Table A2).

Testing TNT and ONT levels within the technique factor using the one-sample *t*-test showed consistent 95% confidence intervals for the magnitude of index pain relief (Table A3); WCA between 82 and 93% in the TNT subgroup, compared to 36 to 69% in the ONT subgroup: BCA between 82 and 93 in the TNT subgroup compared to 48 to 76% in the ONT subgroup: WCA duration of pain relief showed 9–13 months 95% confidence intervals in the TNT group compared to 4–8 months in ONT subgroup: BCA and 6–9 months in the ONT compared to 9–13 months in the TNT subgroup (Table A4).

All four pain relief outliers and all LTF are in the ONT category, thus accounting for the unchanged confidence levels for the TNT (Fig. 2). Outliers include three cases with relief between 25% and 50% and one with no pain relief. Both WCA and NCA plots show outliers denoted by separate red dots before trimming the BCA (Fig. 2).

**Fig. 3:**
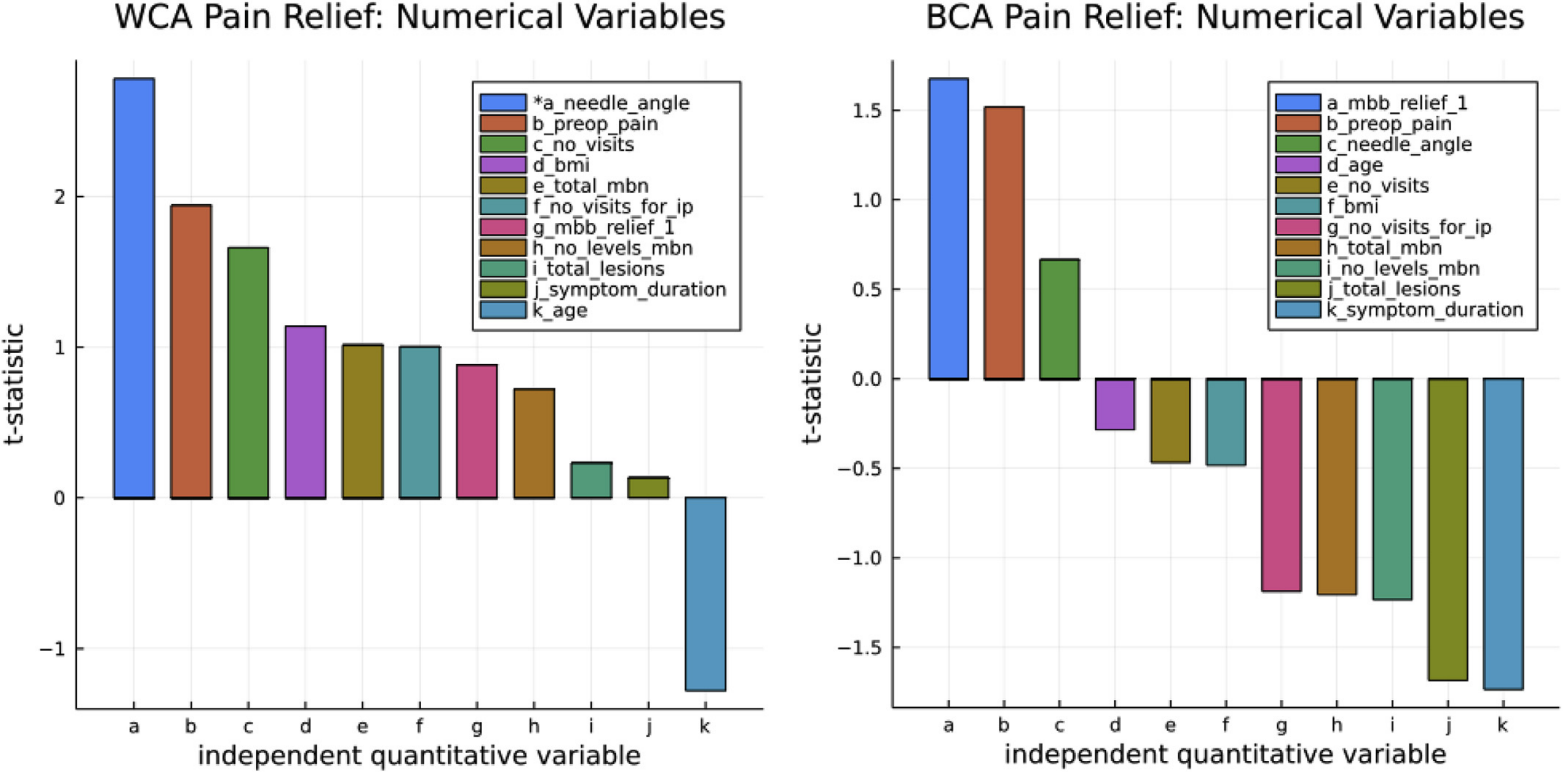
Plots of individual correlation results for BCA using the dependent variable percent of pain relief and eleven independent numerical factors. The t-statistic allows visualization of positive and negative correlations, with more significant differences having greater positive or negative values. Using multiple variable individual testing is prone to type 1 error. We use the test as a qualitative “clinical” screening tool looking for potential confounding variables. The needle insertion angle is measured from 0 to 90° to the transverse process and is statistically significant in the WCA, indicating that a steeper cannula insertion angle may influence outcomes. Note: ip ​= ​no of visits for index pain; bmi ​= ​body mass index; total_mbn ​= ​total primary and repeat TMBNs.

An exploratory multiple factor analysis of independent continuous predictors found no variable showing a statistically significant association with the magnitude or duration of pain relief except for needle angle in WCA. BCA had no continuous independent variables that predicted the magnitude of pain relief (Fig. 3).

**Fig. 4:**
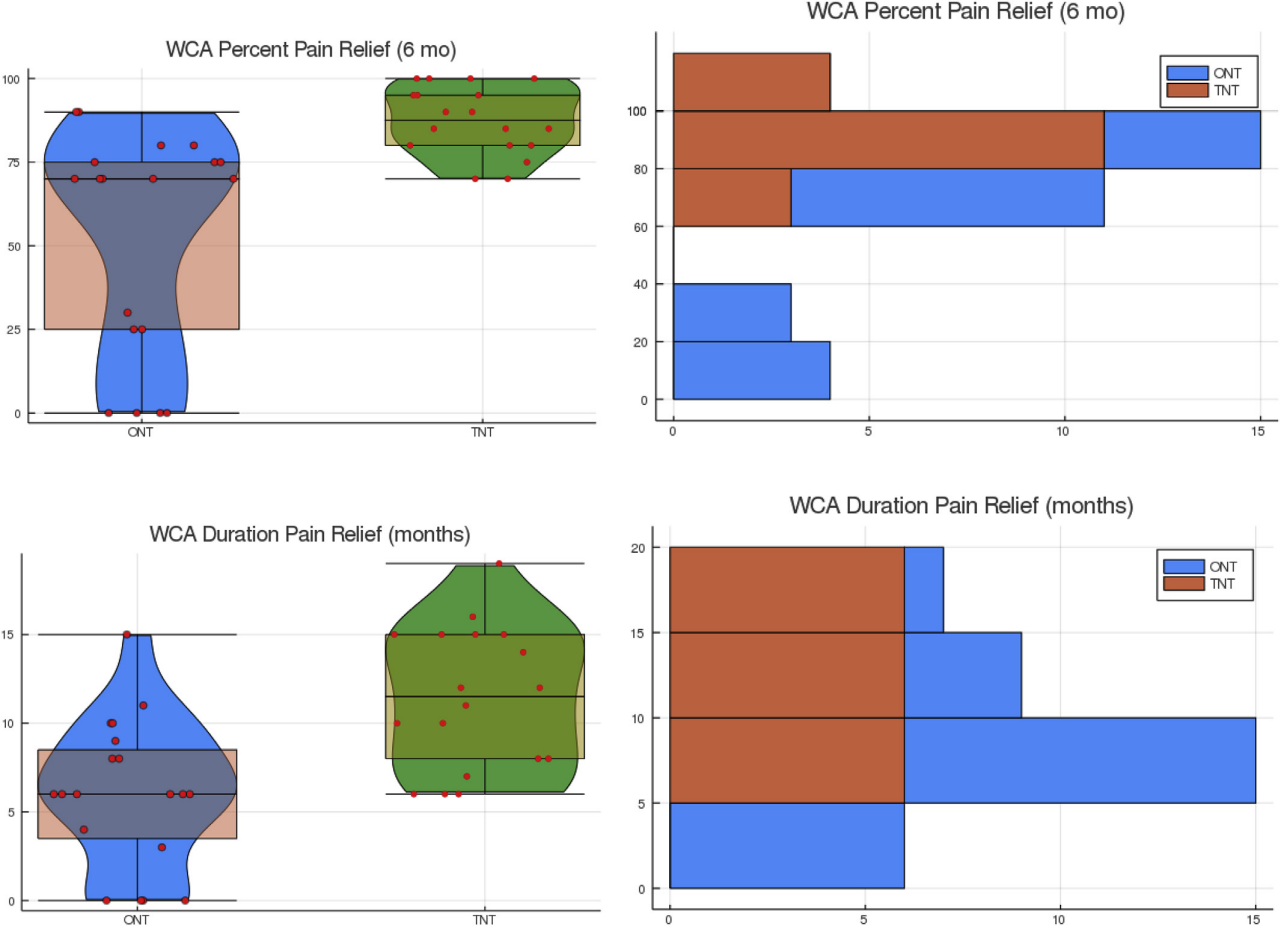
The left figures are violin plots overlayed with a box plot with whiskers that extend 1.5 quartile distance above and below the second and third quartiles. The overlayed red dots represent percent pain relief of individual cases using a WCA, and the “violin” is calculated outcome distributions. The median percent relief and duration of relief are the lines within the respective boxes. Three of the 0% relief are the LTF cases. Note how the percent relief aligns in 5% “categorical” groups. The right plots are the same data in a standard stack bar graph grouped by 20% increments for percent pain relief at six months and five-month increments for duration of pain relief in months. Note that the 100% pain relief bar is for all patients reporting 100% pain relief. (For interpretation of the references to colour in this figure legend, the reader is referred to the Web version of this article.)

The BCA of the OW-ANOVA analysis of independent categorical variables identified several potential confounding factors that might contribute to outcome (Fig. 4).

## 6 Discussion

“Correlation does not imply causation; … Drawing valid causal inferences on the basis of observational data is not a mechanistic procedure but rather always depends on assumptions that require domain knowledge and that can be more or less plausible.” [16].

**Fig. A3:**
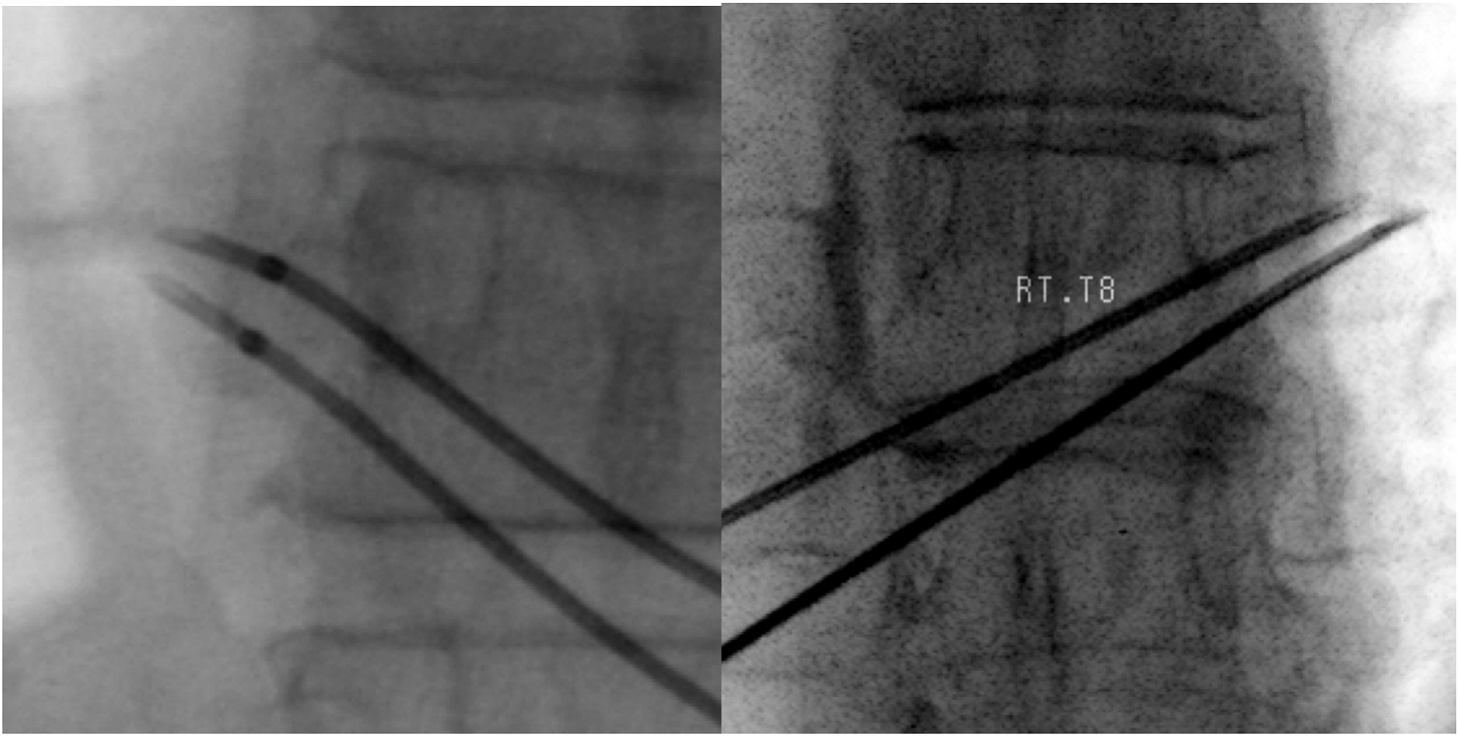
The left image shows an MLA at the T10 level, targeting the medial branch close to the superior lateral edge of the transverse process [13]. The electrodes are at a 45-degree insertion angle, the electrodes ∼5 ​mm apart, which is ∼half the width of the transverse process [15]. At an insertional angle less than ∼60°, one should not nonchalantly advance cannula off the upper edge of the transverse process. The right image is a like example in an older patient with a mild thoracic kyphotic degenerative deformity with multi-segmental pain. The patient reported relief of their worst upper thoracic index symptoms but continued to have pain below the MBN levels.

**Fig. A4:**
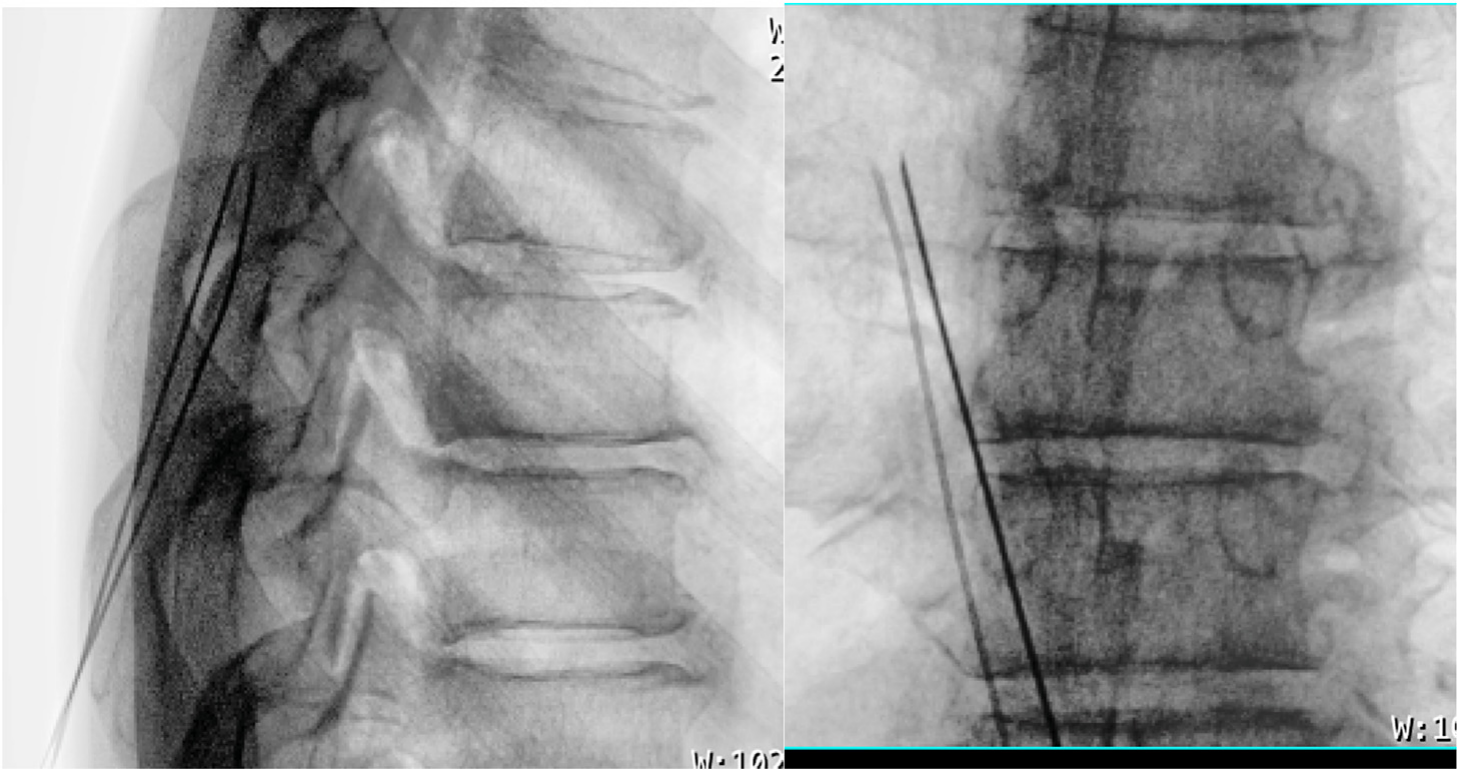
Cadaver images using on-site available 3.5-inch twenty-gauge spinal needles, one straight and one with a slight distal curve. The entrance is one finger breath ipsilateral to the tip of the transverse process and lower endplate one level below the index transverse process allowing a flatter AP angle of insertion. The medial to lateral insertion angle is ∼75°. Note that the tips are safely posterior to the lung and do not extend laterally to the tip of the transverse process. One may consider additional lesions closer to the lateral edge of the transverse process, medially - closer to the facet joint, or withdraw the cannula to place it in a more traditional position over the transverse process.

Acknowledging the challenges of proving causation, we compared thoracic medial branch neurotomy (TMBN) technique in a cohort of consecutive patients. We compared pain relief and duration using two TMBN technical variations performed in a controlled clinical setting over ten years by one interventionalist at one facility in a patient population most referred after failing conservative, interventional, or surgical treatment. The first author used a medial to lateral MBN approach supplemented with mild neurolytic, procedural sessions varying only by the radiofrequency cannula (RFC) angle of insertion that varied from ∼10° parallel to the transverse process ​∼ ​70°. (Fig. 1, A3), and an arguably random choice to use either a single RFC or a single lesion (ONT) versus using two RFCs and performing simultaneous heating (TNT).

The first author typically chose the ONT technique for cases with multiple bilateral painful spine segments and complex anatomy as he perceived that such patients would be more at risk for complications or worsening of their pain caused by the needle trauma and prolonged operative times. TMBN patients were uncommon and understudied, and it was unknown whether the number of lesions could make a clinically significant difference. A genuinely random choice of TNT versus ONT would permit a random-effects model to generalize OW-ANOVA finding to a more general population; in our case, patients referred to an interventional clinic for consultation or procedures [17].

**Fig. A5:**
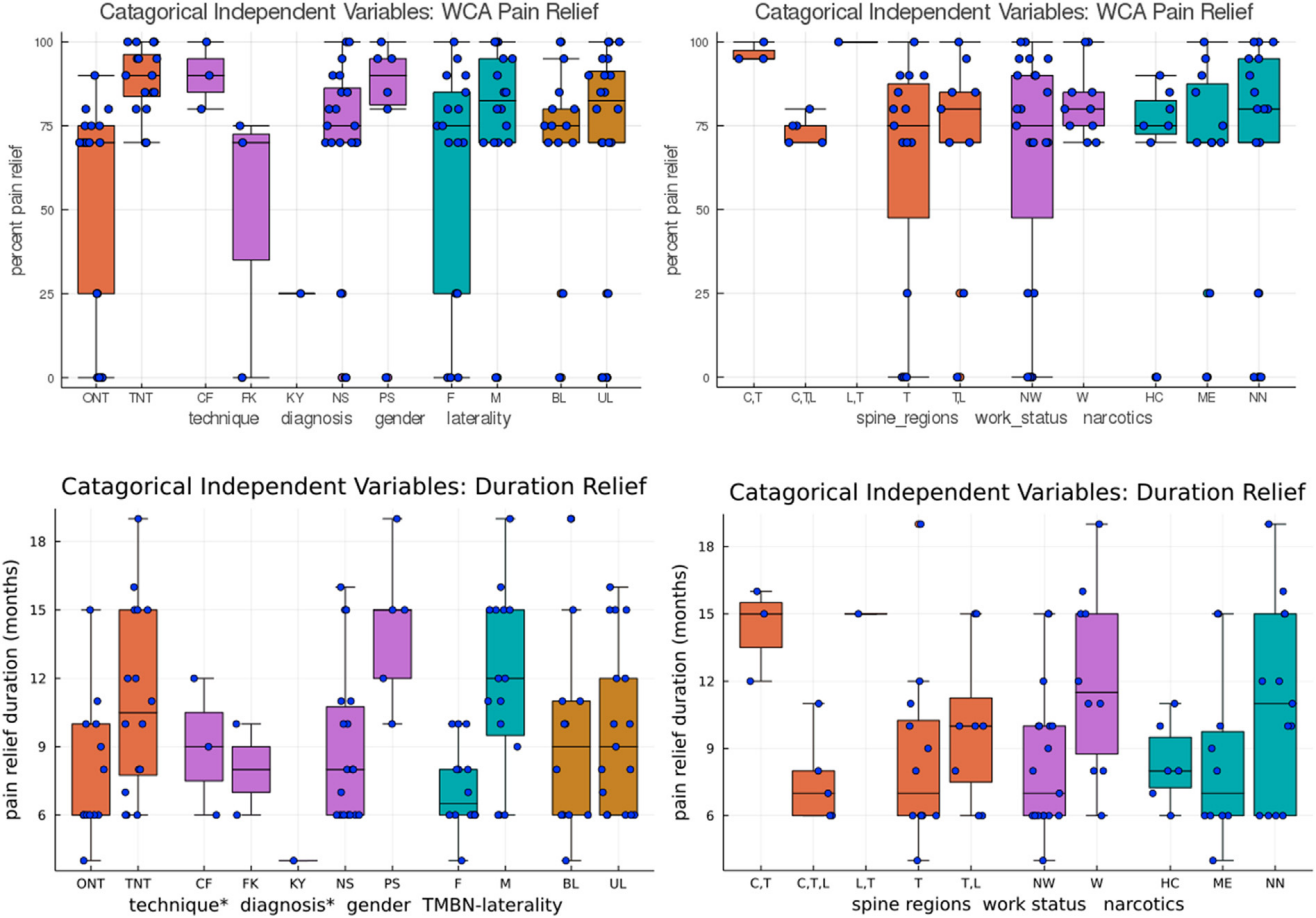
Box and Whisker Plots of WCA categorical independent variables using percent pain relief as the dependent variable top and duration of pain relief bottom.

All three LTF cases and five outliers were in the ONT group (Fig. 2). Whether the four negative outliers resulted from technical failure, diagnostic failure, chance, or a combination is uncertain. More important and supported by our preliminary statistical evaluation of confounding variables, the underlying cause is unlikely a disproportionate spread of unfavorable factors between the ONT and TNT subgroups, although other clinical factors may have affected the overall outcome. Rather than repeating patient demographics listed in our prior paper [11], we evaluated available potential confounding continuous and categorical variables to determine whether any variable had a potentially significant power to predict outcome (Fig. 3, Fig. 4, Fig A5) (Table A5).

Showing a highly significant separation of the TNT from the ONT group is arguably indirect evidence that lesioning the medial to the superior lateral surface of the transverse process produces an effect, as one might expect no difference if a greater lesion size did not improve outcome, and the actual effect mainly due to placebo or dextrose. Whether the additional time and needle trauma of TNT compared to ONT is worthwhile is a case-by-case judgment of the interventionalist. Our study is meant only as a technique to consider.

On the other hand, the comparison between TNT and ONT argues that doing “more” rather than “less” results in better outcomes (Fig. 4). We believe that a prudent clinician should determine the optimal number and size of the lesions for each patient, keeping in mind that “too much” may become harmful. We emphasize that one may achieve expanded lesion size by using variations of the ONT that may achieve an equal or potentially greater heating radius by multiple single unipolar lesions [18] In addition, radiofrequency devices that create a similar uniform expansion of the heat lesion may achieve similar results [19].

Furthermore, one may or may not achieve comparable results using a lateral to medial approach (LMA), and we selected several images to demonstrate the approach (Fig A1). The evolution from the LMA to MLA approach was primarily the result of the first author's perceived safety issues and aspirations for improved outcomes; however, the improved outcomes were most likely achieved due to the new availability of larger gauge radiofrequency needles and the progression to a TNT, both facilitating greater lesion size.

We discussed the primary shortcomings of this study in our recent thoracic outcome study that shared all but the added seven cases for this study [11]. Our former study was a retrospective outcome audit; this study is a comparative study using percent pain relief and duration of pain relief following a patient's first TMBN (except in two cases) as the dependent response variables. However, we again emphasize that our study is a retrospective audit without a control group. There might have been some combination of factors that led the author to use ONT for patients less likely to have good outcomes—particularly selecting the ONT for efficiency reasons for bilateral, multilevel cases. However, statistical testing for the number of levels and the total number of lesions found no statistical evidence of a correlation (Fig. 3).

Further, while the first author consistently dictated the magnitude of pain relief, patient NASS questionaires were not, thus the reason we do not include function outcome. In addition, patients subjectively reported their percent relief to the treating physician, his staff, or both; deficiencies that will artificially increase subjectively reported relief percentage [20,21].

We point out a well-known reality that quantifying subjective pain relief in patients with cyclic segmental axial pain is prone to error and perhaps better evaluated in categorical groups rather than as a continuous variable. Aside from NRS's typical inclusion in outcome studies, a more generalized patient assessment of a percent improvement within ∼10–20% range is perhaps a more practical assessment (Fig. 4) and a reason we used both categorical and continuous statistical assessment (Tables A1-2). Furthermore, there is a quandary of how to categorize outcomes for patients with multiple regional pain sources who obtain relief from their index pain at the location of the MBN but continue to complain of pain at adjacent segments (Fig A3-right).

Although the likely augmented patient-reported results should not significantly affect the comparison, they will affect patient and provider anticipation of improved outcomes using a larger lesion area. As we cautioned in our former study, we again recommend a conservative patient consent, counseling that one has a 50–60% chance of clinically significant pain relief for approximately six months following TMBN using a TNT or a comparative technique with neurolytic supplementation [21,22]. However the data supports those interventionalists using an enthusiastic approach more leeway to quote higher percentages [21].

**Fig. 5:**
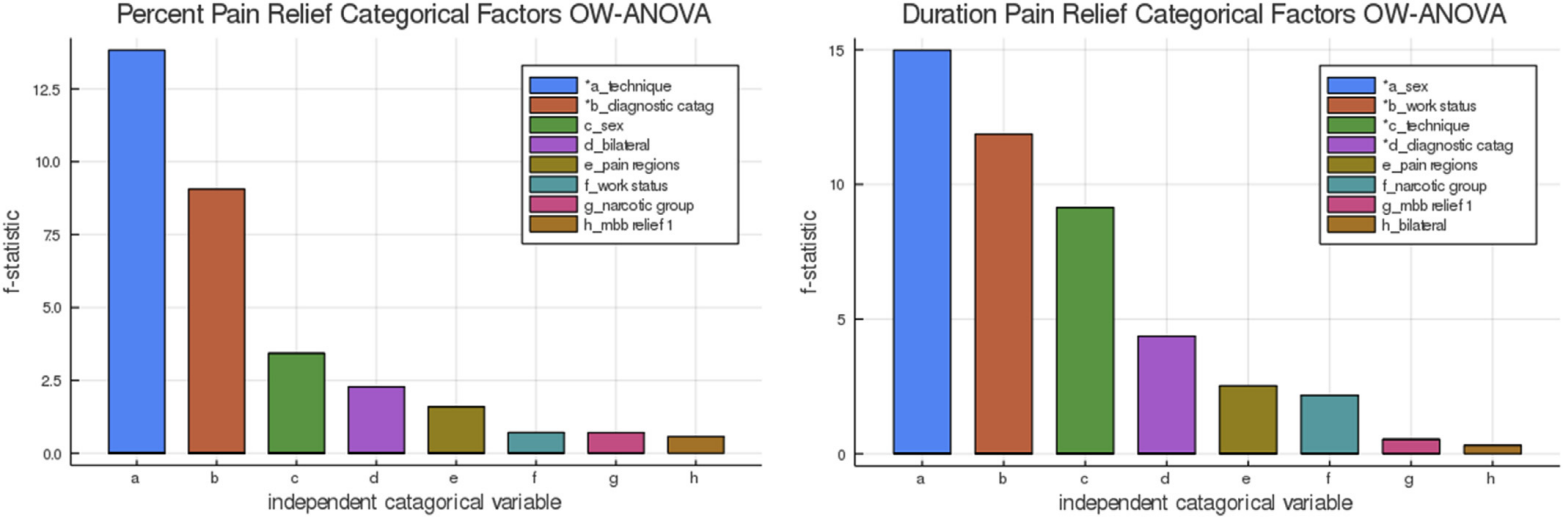
OW-ANOVA of BCA of each potential confounding categorical factor. The larger the f-statistic, the more likely one or more subgroup means are significant. The stared factors have a p-value ≤ .05 that typically requires an f-statistic > 3. Using the Kruskal Wallis Test, the nonparametric screening test of all variables together did not find any independent group with a significance p-value ≤ .05. On the other hand, the duration of pain relief has several factors that might influence the patient-reported duration of pain relief. However, gender is a spurious result. No factor contained more than five levels or subgroups/levels. The appendix contains the results of the OW-ANOVA for BCA of percent pain relief (Table A5) and provides the name of the subgroups. Box and whisker plots of BCA of the categorical variables provide readers with a qualitative view (Fig A5).

We explore but do not detail the confounding effects of other clinical and technical variables on the magnitude and duration of index pain relief—the relatively few cases making regression modeling unreliable (Table A5). We include the resulting plots from correlation testing of potential confounding factors, finding a possible correlation using cannulae angle of insertion in the WCA but not BCA (Fig. 3, Table A5) and the diagnostic category group in BCA that is probably spurious due to few cases in multiple subgroups (Fig. 5). Exploratory evaluation using OW-ANOVA for duration of pain relief found two probable spurious and two potential predictors using the pain duration response variable (Fig. 5 right).

Although included as a confounding variable and of particular interest to interventionalists, we do not explore the predictive value of thoracic MBB in this paper; however, noting that in the specific case of TMBNs, a reasonably safe procedure with robust results, the recently published ASRA lumbar consensus lumbar MBB guidelines recommendations are a reasonable diagnostic protocol for TMBNs [23]. Nevertheless, until a more detailed analysis of the thoracic MBB data is published, when to use higher standards for MBB success and a confirmatory MBB should be a physician-patient judgment based on a patient's circumstances [4,24].

We further remind the readers that the first author used dextrose in approximately equal volumes in both the ONT and TNT techniques, and therefore, the added dextrose should not influence the evaluation of variance or the outcome comparison. Dextrose's effect on outcome is unknown.

Finally, despite the paraphrased opening quote that assumes the results of TNMN depend on lesioning the medial branch, recent anatomical studies support consideration of other reasons for pain relief in the T4 through T8 segments [14]. The apparent better overall degree and duration of relief using the TNT for TMBN compared to reported lumbar MBNs is consistent with a possible additional non-neurolytic effect of heat and dextrose [4,12,[25], [26], [27]]. Still, a mechanism that possibly includes a “regenerative-desensitizing” effect, a possible ablation of entrapped nerves, or denervation of the facet or its capsules should not impune a comparative study's findings. Even so, any nonrandomized and uncontrolled study's placebo effect or patient pain that regresses to a lower mean value by factors other than the treatment is an alternate explanation for subjectively reported pain relief [21].

## 7 Conclusion

TMBN performed with simultaneous lesions using two RF cannula may provide a more clinically significant magnitude and possibly more prolonged pain relief than creating a single lesion with one electrode.

## Conflict of interest

The authors declare that they have no known competing financial interests or personal relationships that could have appeared to influence the work reported in this paper.

## Figures and Tables

**Table A1:** Worst-case (WCA) and best-case (BCA) using one-way analysis of variance (OW-ANOVA) test. ANOVA is a parametric test. The independent predictor or explanatory variable that we named technique consists of the subgroups or levels TNT and ONT, pain relief (PR) the response-dependent variable. The very low p-values indicate that the TNT and ONT are extremely unlikely to come from the same populations.

OW-ANOVA Hypothesis Test: Percent Pain Relief: ONT and TNT Independent Variables					
result	test	f-statistic	p-value	degrees freedom	observations
reject	OW_ANOVA using WCA	18.01464	0.00015	(1, 35)	[18,19]
reject	OW_ANOVA using BCA	11.22032	0.00232	(1, 28)	[12,18]

**Table A2:** Worst-case (WCA) and best-case (BCA) OW-ANOVA test using duration of relief as the dependent variables. Again, the p-value shows it is highly improbable that the TNT and ONT come from the same population.

OW-ANOVA Hypothesis Test Duration Pain Relief: ONT and TNT					
result	test	f-statistic	p-value	degrees freedom	observations
reject	OW_ANOVA using WCA	16.09122	0.0003	(1, 35)	[18,19]
reject	OW_ANOVA using BCA	9.13577	0.00499	(1, 31)	[15,18]

**Table A3:** One-sample T-Test of pain relief degree (PR) using categorical technique factor as the independent predictor or explanatory variable. The separation in the groups is due to the statistical and arguably significant clinical differences in their means with reasonably tight confidence intervals in the TNT group.

One-Sample T-Test: Percent Pain Relief: Technique Categorical Group							
result	mean	name	p-value	count	std dev	low ci	high ci
reject	87.5	WCA technique TNT	1.39E-17	18	10.2	82.4	92.6
reject	52.37	WCA technique ONT	2.39E-06	19	33.7	36.1	68.6
reject	87.5	NCA technique TNT	1.39E-17	18	10.2	82.4	92.6
reject	62.19	NCA technique ONT	1.22E-07	16	26.6	48	76.4
reject	87.5	BCA technique TNT	1.39E-17	18	10.2	82.4	92.6
reject	76.25	BCA technique ONT	1.04E-12	12	7.4	71.5	81

**Table A4:** One-sample T-Test for TNT and ONT duration of relief (DR) The separation in the groups is due to statistical and arguably clinical differences in their means with less tight confidence intervals than in the (PR) response variable.

result	mean	name	p-value	std dev	low ci	high ci
reject	11.39	WCA technique TNT	9.16E-10	4	9.4	13.4
reject	6	WCA technique ONT	6.36E-06	4.2	4	8
reject	11.39	NCA technique TNT	9.16E-10	4	9.4	13.4
reject	7.12	NCA technique ONT	6.92E-07	3.5	5.3	9
reject	11.39	BCA technique TNT	9.16E-10	4	9.4	13.4
reject	7.6	BCA technique ONT	1.41E-07	3	5.9	9.3

**Table A5:** An example using best-case analysis (BCA) of pain relief of our one-way ANOVA Test, the BCA removing lost to follow-up (LTF) and trimming outliers. We sorted the table according to the f-statistic (and p-value) for independent categorical groups with two or more categories. Two rejected categories, gender and work status in the WCA, failed rejection (unlikely predictive) after trimming. Although the ANOVA test rejected the diagnostic group null hypothesis, both patients in the TK category (thoracic kyphosis) happened in the one needle group, and both were low pain relief outliers having a p-value. The p-value of the technique group is five times lower than the diagnosis group. Also, note that MBB relief, tested as a categorical variable, is almost significant at the .05 and therefore may have modest to strong predictive value. One may note the number of observations in each group (nobs), several levels having only two observations. The subgroups would need further consolidation to analyze two predictive factors.

BCA OW-ANOVA of Categorical Independent Variables for Percent Pain Relief					
result	F-statistic	category and subgroup names	p-value	deg freedom	no observation
reject	13.8288	technique ONT: one needle technique TNT: two-needle technique	0.00079	(1, 31)	[15,18]
reject	9.05865	diagnostic category CF: compression fracture FK: failed [kyphoplasty] NS: nonspecific DDD, disc, myofascial PS: post [T] spine fusion KY: kyphosis	8.00E-05	(4, 28)	[[2], [3], [4], [5],^19^]
fail	3.42354	gender M: male F: female	0.07382	(1, 31)	[16,17]
fail	2.2762	procedure laterality UL: unilateral BL: bilateral	0.1415	(1, 31)	[14,19]
fail	1.58884	mbb_relief	0.21425	(3, 28)	[3,5,9,15]
fail	0.70612	work status working not working	0.40738	(1, 30)	[11,21]
fail	0.70359	narcotic group ME: morphine equivalent. HC: hydrocodone equivalent NN: no narcotics	0.50279	(2, 30)	[8,11,14]
fail	0.56507	mbb relief	0.77532	(7, 20)	[2,2,3,3,3,3,4,8]
